# Regression Analysis and Comparison of Economic Parameters with Different Light Index Models under Various Constraints

**DOI:** 10.3390/s21227561

**Published:** 2021-11-14

**Authors:** Debao Yuan, Huinan Jiang, Wei Guo, Ximin Cui, Ling Wu, Ziruo Wu, Hongsen Wang

**Affiliations:** School of Geoscience and Surveying Engineering, China University of Mining and Technology (Beijing), Beijing 100083, China; yuandb@cumtb.edu.cn (D.Y.); weiguo@cumtb.edu.cn (W.G.); cxm@cumtb.edu.cn (X.C.); SQT2100204089@cumtb.edu.cn (L.W.); ZQT1900204091g@cumtb.edu.cn (Z.W.); ZQT2000204104@cumtb.edu.cn (H.W.)

**Keywords:** VIIRS-DNB, night lights, conditional constraint, *VHNI*, linear regression

## Abstract

Economic globalization is developing more rapidly than ever before. At the same time, economic growth is accompanied by energy consumption and carbon emissions, so it is particularly important to estimate, analyze and evaluate the economy accurately. We compared different nighttime light (*NTL*) index models with various constraint conditions and analyzed their relationships with economic parameters by linear correlation. In this study, three indices were selected, including original *NTL*, improved impervious surface index (*IISI*) and vegetation highlights nighttime-light index (*VHNI*). In the meantime, all indices were built in a linear regression relationship with gross domestic product (GDP), employed population and power consumption in southeast China. In addition, the correlation coefficient $R^{2}$ was used to represent fitting degree. Overall, comparing the regression relationships with GDP of the three indices, *VHNI* performed best with the value of $R^{2}$ at 0.8632. For the employed population and power consumption regression with these three indices, the maximum $R^{2}$ of *VHNI* are 0.8647 and 0.7824 respectively, which are also the best performances in the three indices. For each individual province, the *VHNI* perform better than *NTL* and *IISI* in GDP regression, too. When taking employment population as the regression object, *VHNI* performs best in Zhejiang and Anhui provinces, but not all provinces. Finally, for power consumption regression, the value of *VHNI* $R^{2}$ is better than *NTL* and *IISI* in every province except Hainan. The results show that, among the indices under different constraint conditions, the linear relationships between *VHNI* and GDP and power consumption are the strongest under vegetation constraint in southeast China. Therefore, *VHNI* index can be used for fitting analysis and prediction of economy and power consumption in the future.

## 1. Introduction

To better understand global change, its causes and its implications, we can relate human activities to natural physical quantities. As an important part of global change, it is necessary to study economic change. Human activity is not simply distributed uniformly across the earth’s surface, but its distribution trajectory is affected by many factors, such as rivers, altitude, light and others. The characteristic of human activities is concentration and aggregation, so it is easy to identify the activity track of human society according to this characteristic. As one of the unique features of human society, light recognition can identify the gathering place of human society.

Nighttime light (*NTL*) data are used as medium resolution images to explore human social activities [1], such as lighting area [2], population [3,4,5], economy [6,7,8], built area [9], power consumption [10,11], and have achieved good results. The study of the relationship between lighting data, population and gross domestic product (GDP) has also had good results [12,13,14]. At the same time, the relationship between lighting data and population is also tested on a small scale [15]. Therefore, light data can be a good method for socioeconomic estimates. There are two types of nighttime light data most commonly used today, one is the U.S. Air Force’s Defense Meteorological Satellite Program (DMSP) Operational Line Scan System (OLS) and the other one is the Suomi National Polar-Orbiting Partnership (NPP) Visible Infrared Imaging Radiometer Suite (VIIRS) with Day/Night Band (DNB). VIIRS sensor data has higher spatial resolution than DMSP-OLS. What is more remarkable is that VIIRS has a strong ability to amplify low-brightness visible light related to human activities and can observe cities, fishing boats and gas firelight at night without background moonlight [16,17]. Therefore, compared with DMSP-OLS, VIIRS-DNB can provide richer information about human habitation and economic activities [18]. Due to the defects of OLS sensors themselves, night light data in urban centers with high light intensity will show the light saturation phenomenon, that is, DN value increases to a certain extent and does not continue to increase with the increase of ground light intensity.

In view of the shortcomings of traditional studies, Tang et al. took DMSP/OLS light data as the data source, which could better reflect the economic development level of provinces [19]. Using Zimbabwe as the study area, the correlation between total night-time lighting (*TNL*) and GDP as an indicator, Li et al. compared nighttime lighting images of four regional countries and regional scales in 2000 and 2008, and found that Zimbabwe’s economy declined between 2000 and 2009. Chen. J compared moderate resolution imaging spectroradiometer (MODIS) data with DMSP/OLS and NPP/VIIRS nighttime light data to estimate provincial economic development, and found that, compared with DMSP/OLS nighttime light data, MODIS data improved the accuracy of estimation [20]. In addition, MODIS data are more accurate than DMSP/OLS nighttime light estimates, especially in dark and light-saturated areas, but not as accurate as NPP/VIIRS nighttime light estimates. Taking the U.S. states and metropolitan statistical area for comparison, Chen found VIIRS data in the prediction of cross section is more useful than forecast time series data [21].

Omurakunova studied the effectiveness of night light (*NTL*), a data source, in monitoring factors related to sustainable development in the Bangladeshi environment [22]. In 2017, Fu put forward the economic index of night lighting (NLEI), and conducted correlation analysis of NLEI and economic indicators of various provinces and cities in China from 1992 to 2012 by using DMSP/OLS data [23]. Qun used the vegetation-adjusted *NTL* urban light index (VANUI) to alleviate the light overflow problem of DMSP data in big cities. Thus, the dynamic change of urban information systems in China from 1992 to 2009 was estimated. Wei. G utilizes improved impervious surface index, (*IISI*) estimated the impervious water surface area of arid/semiarid urban areas through linear regression model [24].

It can be seen that the research methods of night light and economy mostly take economy as the index to fit with light data, and most of them are improved in the fitting method. Most of the light indices proposed by scholars, such as *IISI* and VANUI, focus on impervious water surface and other economy-related studies, without direct and effective studies on the economy itself. Therefore, this paper aims to put forward a light index fitting with the economy and make a comparative analysis with other indices.

## 2. Materials and Methods

### 2.1. Study Area

**Figure 1:**
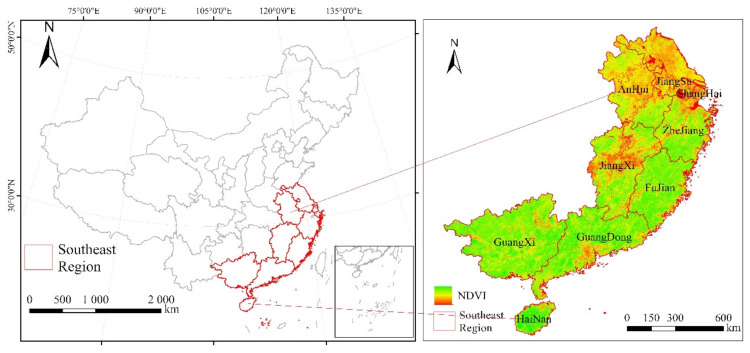
Study area including eight southeast provinces and Shanghai. The figure on the left shows the location of the study area and the right figure shows the location image of the study area based on *NDVI*.

With the exception of Hong Kong, Macao and Taiwan province, China’s major economic regions are also divided in different ways. In addition to their geographical differences, these regions also widely reflect the overall differences in social and economic development. Since the beginning of China’s reform and opening up, southeast China’s natural geographical advantages have led to its rapid economic development. With the introduction of various policies and benefits, southeast China has become the most developed region in China’s economy. The Yangtze River Delta and Pearl River Delta economic zones are the most active regions in China’s economic development. The economic conditions of the southeast region are very good as it hosts Shanghai, China’s economic center and national metropolis, and Shenzhen, a special economic zone in the region. Therefore, this paper chooses the economically and culturally developed southeast region as the research area (Figure 1). The southeast region, located in the southeast of China, includes Jiangsu, Shanghai, Zhejiang, Fujian, Jiangxi, Anhui, Guangdong, Guangxi and Hainan (excluding Sansha City) (Table 1).

Four kinds of data were used in this study, as shown in Table 2, including NPP/VIIRS 2015 annual data [25] and MODIS normalized difference vegetation index (*NDVI*) data. Administrative boundaries and socioeconomic statistics of provinces, municipalities and prefecture-level cities.

### 2.2. Data Preprocessing

The VIIRS-DNB data are on a geographic (lat/lon) grid with cell size 15 arc-seconds. With the increase of latitude, the image grid footprint increases. In order to avoid the influence of image grid deformation on nighttime image data and facilitate statistical information, nighttime light images of China’s administrative regions were first extracted. We reprojected the extracted images to the Albers projection and resampled the data to the size of 500 m × 500 m cells using the nearest neighbor algorithm. The annual product was further processed to remove background noise. However, there were still some extremely high light values, such as those of airport lights and power plants. In order to reduce these influences, some studies use the threshold method to remove background information and images [26]. The values for all core areas of the city were below 250 nW cm^−2^sr^−1^ because the threshold was set to the maximum, that is, all pixel values over 250 in the study area were reassigned to 250.

### 2.3. Light Index Model

Considering that the study area is mainland China and there are great differences in land cover types and vegetation coverage among different areas, two types of indices were selected, namely *NTL* index without other restrictions, and improved impervious surface index (*IISI*) of weak light area enhanced by logarithm. They can be represented separately by the formula:

(1) NTL=DNB

*NTL* stands for preprocessed data of VIIRS-DNB, and *DNB* is the pixel value of the corresponding pixel.

(2) $$DNB_{nor}=\frac{DNB-DNB_{min}}{DNB_{max}-DNB_{min}}$$

where $DNB_{nor}$ represents the normalized VIIRS-DNB image value range from 0 to 1, $DNB_{min}$ is the minimum value and $DNB_{max}$ the maximum value, which were 0 and 200, respectively.

(3) $$IISI=log_{2}\sqrt{1+DNB_{nor}}$$

where $DNB_{nor}$ represents normalized VIIRS-DNB value. The logarithm model and coefficient can guarantee an index value between 0 and 1. This index not only suppresses extremely large values but also enhances small ones.

Considering that vegetation has a negative correlation with human activities, that is, the intensity of human nonagricultural activities in the central area of the city is high, and the vegetation coverage is generally less. However, in rural areas, the intensity of human nonagricultural activities is low and vegetation coverage is high. The *NTL* value of light intensity decreases gradually from the urban center to the outskirts, while the *NDVI* shows an almost opposite trend. After *NTL* is normalized to get $DNB_{nor}$, it is taken square treatment to achieve the effect of enhancing light intensity.

Due to the nature of the square root function itself, the closer the value of $DNB_{nor}$ is to 1, that is, the closer it is to the city center, the weaker the enhancement effect is, while the closer the value of $DNB_{nor}$ is to 0, the farther away from the city center, the stronger the enhancement effect is. When $\sqrt{DNB_{nor}}$ is equal to *NDVI* and the value of $DNB_{nor}$ is less than *NDVI*, which usually occurs in the rural-urban transition area and the countryside far away from the city center, so the $\sqrt{DNB_{nor}}$ is equal to *NDVI* is taken as the reference. Set the adjustment coefficient of *NTL* to 1 at this time to achieve the role of light value in the prominent area. When $\sqrt{DNB_{nor}}$ is greater than *NDVI*, the larger the difference value is, the greater the light intensity is and the closer it is to the active light area. When $\sqrt{DNB_{nor}}$ is less than *NDVI*, the larger the difference value is, the weaker the light intensity is. Therefore, when $\sqrt{DNB_{nor}}$ is larger than *NDVI*, the spatial differences of *NTL* within the region are enhanced, and the adjustment coefficient of *NTL* is greater than 1. When $\sqrt{DNB_{nor}}$ is less than *NDVI*, the adjustment coefficient of *NTL* is set to be less than 1 in order to enhance the difference of light intensity between suburban and rural areas and city center. Based on this idea, the light index shown in Formula (4) is as follows:

(4) $$VHNI=(\frac{1+\sqrt{DNB_{nor}}}{1+NDVI})*NTL$$

where *VHNI* is the improved lighting index. Theoretically, when $\sqrt{DNB_{nor}}$ is equal to *NDVI*, the value of *VHNI* remains unchanged. When $\sqrt{DNB_{nor}}$ is larger than *NDVI*, the maximum theoretical value is two times *NTL*. When $\sqrt{DNB_{nor}}$ is less than *NDVI*, the maximum theoretical value is 0.5 times *NTL*. Under the condition of *NTL* data pretreatment, the calculated *VHNI* will not have infinite or infinitesimal outliers.

The *NTL* model is preprocessed light data. Because noise removal and extremely high value elimination will not affect the distribution characteristics of DN value of light data, normalization of *NTL* will not cause abnormal influence. Therefore, both *NTL* and $DNB_{nor}$ can be defined as unrestricted light index.

*IISI* index is according to Formulas (2) and (3), the normalization processing of lighting data itself only adjusts the range of DN value of lighting data and distributes it in the interval of [0,1]. However, *IISI* index adopts the logarithm function model. As it is a concave function, the closer the independent variable is to the right range within the scope of definition, the slower the growth rate of the value of the corresponding dependent variable is, the DN value of the light data will be constrained, and the original distribution characteristics and laws will change. Therefore, it is defined as a single light restricted light index.

The vegetation represented by *NDVI* is added to *VHNI*, and the lighting type is *NTL* model, but its adjustment coefficient $\frac{1+\sqrt{DNB_{nor}}}{1+NDVI}$ represents not a simple “non-negative is positive”, but highlights the lighting area or vegetation covered area by comparing the lighting value with vegetation. The appearance of $\sqrt{DNB_{nor}}$ makes the original unrestricted light index (*NTL* or $DNB_{nor}$) become a single light limiting index. After the introduction of vegetation factor, the adjustment coefficient of *NTL* becomes complicated, so it is defined as the light limiting index under vegetation constraint.

**Figure 2:**
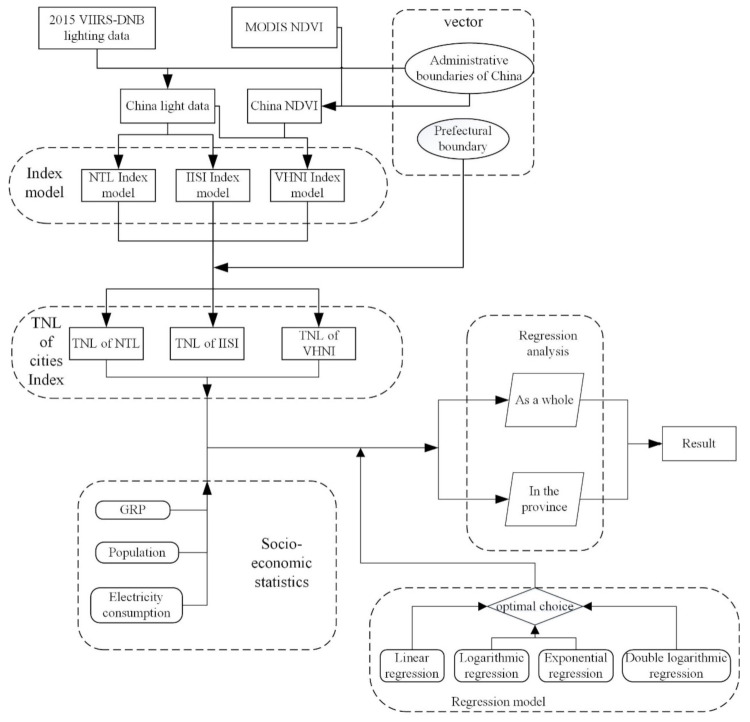
Flowchart. Comparison of regression analysis between different models and three economic parameters.

Firstly, the nighttime light data and *NDVI* data of China were obtained by using the preprocessed light data and MODIS *NDVI* data after clipping the administrative boundary of China. Then, the administrative boundary of prefecture-level cities was cut out and processed to obtain the lighting data of three models, and the *TNL* values of the lighting data of three models in different cities were statistically obtained. The flow chart is shown in Figure 2.

### 2.4. Rregression Model

We chose four regression models which are the most commonly used in regression analysis to evaluate the potential of nighttime light data for modeling socioeconomic parameters [27,28,29,30,31]: linear regression model (Equation (5)), logarithm regression model (Equation (6)), exponential regression model (Equation (7)), and double logarithm model used for those with small dependent and independent variables (Equation (8)):

(5) $$P=a*TNL_{X}+b$$

(6) $$P=a*ln_{TNL_{X}}+b$$

(7) $$P=a*e^{TNL_{X}}$$

(8) $$ln\left(P\right)=a*ln\left(TNL_{X}\right)+b$$

*P* represents social and economic parameters (GDP, power consumption and population), TNL is the total night light intensity of each administrative region (i.e., the sum of all pixel values of light data within the administrative region), $TNL_{X}$ is the total night light intensity of different light index models (*NTL*, *IISI*, *VHNI*).

According to the performance of the four regression models in the overall regression of different indicators in southeast China, which regression model to use in the regression analysis was decided.

## 3. Result

### 3.1. Exponential Comparative Analysis

**Figure 3:**
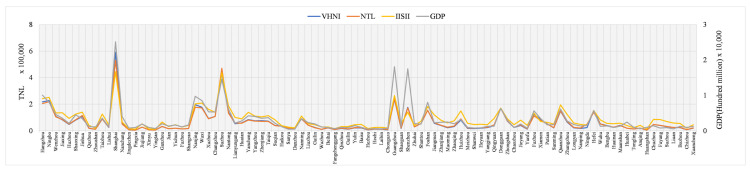
*TNL* and GDP municipal scale of different indices.

**Figure 4:**
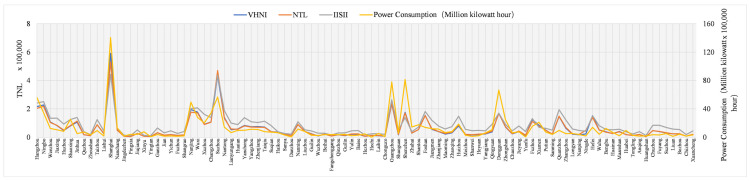
*TNL* of different indices and power consumption at municipal level.

**Figure 5:**
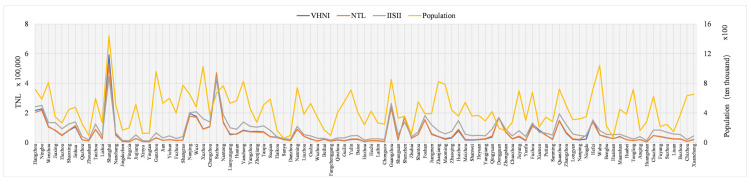
*TNL* of different indices and population at municipal level.

The research area of this paper is the southeast region, where the regional economy is developing rapidly, especially in the provincial capital cities. However, the economic development gap between cities in the study area is still large, there are still some cities in the province lagging behind in economic development. Therefore, in order to understand the relationship between different indices and indicators, the performance of different indices on the same index in different cities was compared. The comparison results are shown in Figure 3, Figure 4 and Figure 5. In Figure 3, Figure 4 and Figure 5, the horizontal coordinate is city, the left ordinate is *TNL*, and the right ordinate is GDP (100 million yuan), population (ten thousand people) and power consumption (ten thousand KWH). Due to logarithm processing, *IISI* index has a small value, so it needs to be magnified by 50 times to unify the order of magnitude of the three indicators.

As can be seen from the GDP curve in Figure 3, except Guangdong and Fujian, provincial capitals are all the economic centers in other provinces, making their economies come out on top. Guangdong province, with Guangzhou and Shenzhen as the centers, has developed in parallel. Fujian province’s Fuzhou, Quanzhou two economic development. In the power consumption curve shown in Figure 4, Shanghai has the largest power consumption, while Guangzhou, Shenzhen and Fuzhou have similar power consumption. Except Guangdong province, the capital cities of different provinces have the most power consumption in their own province. Opposite to GDP and power consumption is the population in Figure 5. Shanghai has the largest population and far exceeds that of other cities, while the population of other cities in the same provinces has little difference.

The curve in Figure 3 and Figure 4 shows that the trend of different indices, GDP and power consumption in different cities is basically the same. The values of the three indices in different cities are slightly different and the trend is basically the same. It is worth noting that, compared with GDP, *VHNI* and *NTL* are very similar in most cities, but there are quite different values in Hangzhou, Wenzhou, Jinhua, Bozhou, Anqing, Chuzhou, Zhanjiang, Zhaoqing, Wuhu and Huaibei, and the trend is opposite. In comparison with power consumption, the trend of the three indices in Hangzhou and Wuhu is opposite to the actual situation of power consumption, and the trend of the other places is consistent. As can be seen from Figure 5, the population and the three indices have a great contrast among different cities, and the trend is opposite in many places such as Hangzhou, Wuxi, Liuzhou, Huaibei and Bozhou.

### 3.2. Regression Analysis in General

**Figure 6:**
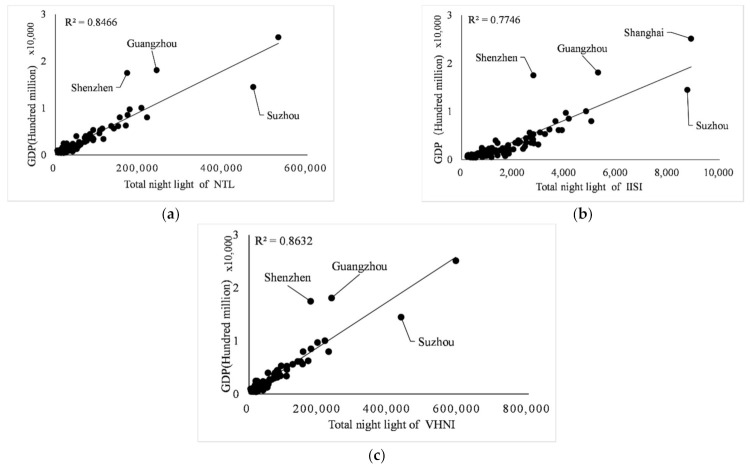
(**a**–**c**) Linear fitting relationships between *NTL*, *IISI*, *VHNI* and GDP, respectively.

**Figure 7:**
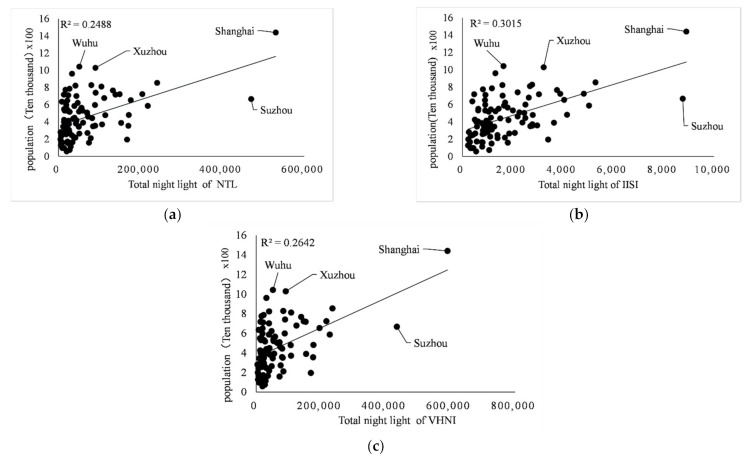
(**a**–**c**) Linear fitting relationships between *NTL*, *IISI*, *VHNI* and population respectively.

**Figure 8:**
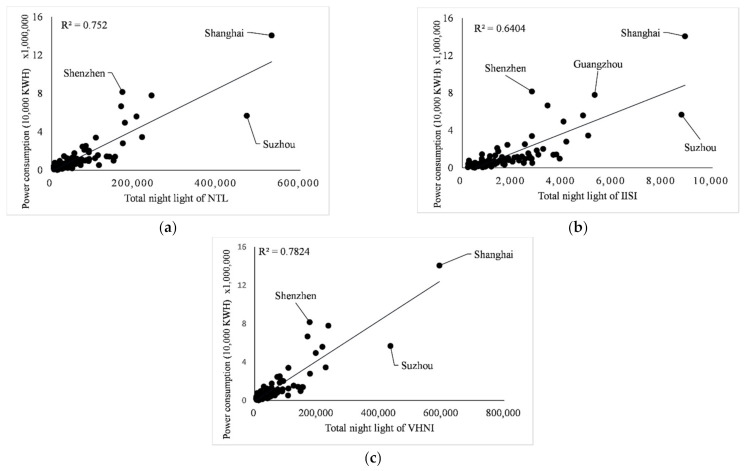
(**a**–**c**) Fitting relationships between *NTL*, *IISI*, *VHNI* and power consumption, respectively.

Taking each city as a part of the whole, linear regression, logarithm regression, exponential regression, and double logarithm regression were carried out for the three indicators. The gross domestic product includes the gross domestic product (including urban population) of prefecture-level cities within each province, in the unit of 100 million yuan. The regression of GDP, population and power consumption of 100 cities in eight provinces and one city in southeast China was carried out through *TNL* of three light indices. The determination coefficient $R^{2}$ of linear, logarithm, exponential and double logarithm model is shown in Table 3. The fitting results of the three indices are shown in Figure 6, Figure 7 and Figure 8.

It can be seen from Table 3 that in the regression analysis of the three indicators of all municipal administrative regions, in the regression analysis of GDP, population and power consumption by linear regression model, the value of $R^{2}$ is almost the highest among the four models, except 0.3015 is less than the 0.3059 of the double logarithm in regression of *IISI* and population. Therefore, for consistency of regression models and strong linear relationship between indicators and indices, only the linear regression model is used for regression analysis below.

In the linear regression analysis results, the index with the best comprehensive performance of the three index fitting results is GDP, and its determination coefficient $R^{2}$ is the maximum in the linear fitting of different indices to the same index, which is 0.8466 for *NTL*, 0.7746 for *IISI* and 0.8632 for *VHNI*, respectively. The indicator with the worst fitting performance was the population, and the determination coefficient $R^{2}$ was the minimum of the three indicators, which were 0.2488 for *NTL*, 0.3015 for *IISI* and 0.2642 for *VHNI*. In the fitting with GDP as the dependent variable and light index as the independent variable, light index *VHNI* performed best, $R^{2}$ of linear regression was 0.8632, higher than 0.8466 of *NTL*, and *IISI* performed worst, $R^{2}$ was 0.7746. In the fitting of population size, the linear relationship between *TNL* and population size of each index is weak, and the value of $R^{2}$ is generally low. The $R^{2}$ of the three indices is about 0.30, but *IISI* is more outstanding, for which $R^{2}$ is 0.3015, which is greater than the 0.2488 for *NTL* and 0.2642 for *VHNI*. In the fitting of power consumption, *IISI* has the worst performance with $R^{2}$ of 0.6404, while *VHNI* has the best performance with $R^{2}$ of 0.7824, which is larger than 0.752 of *NTL*. As can be seen from Table 3, in the fitting of GDP and power consumption, the determination coefficient $R^{2}$ of *VHNI* has the highest value and the strongest linear relationship, which is greater than the other two indices.

In the case of poor fitting relationship between population quantity and *TNL* index in southeast China, this paper takes into account social factors in southeast China, the relationship between age and labor force, and uses urban employed population to replace local population quantity as an indicator to make linear regression again. The regression determination coefficient $R^{2}$ is shown in Table 4.

**Figure 9:**
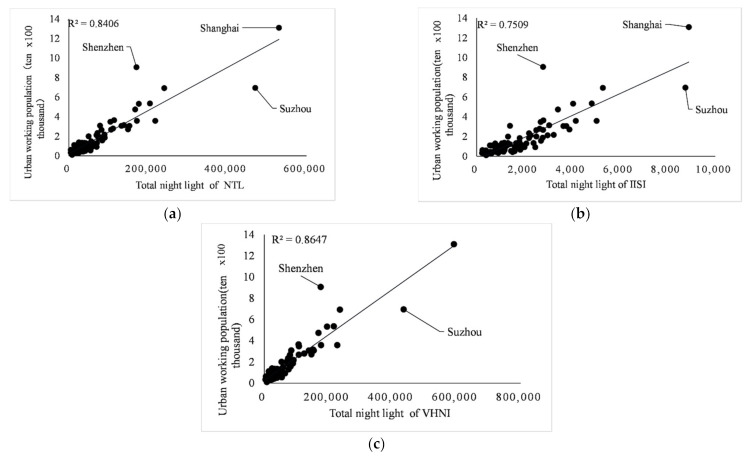
(**a**–**c**) Fitting relationships between *NTL*, *IISI*, *VHNI* and urban employed population respectively.

By comparing Table 2 and Table 3, it is found that the determination coefficient $R^{2}$ of each index is significantly improved after the urban employed population replaces the total population index through linear regression, and the fitting degree is good. The determination coefficient $R^{2}$ of *VHNI* is 0.8647, which is still the best among the three indices. Southeast China has a developed economy, large population and complex structure, and many social factors lead to a large number of idle people. After excluding this factor, urban employment population is used as an indicator for fitting. The linear relationship is shown in Figure 9. The regression coefficient $R^{2}$ of *NTL* index is increased from 0.2488 to 0.8406, and the regression coefficient $R^{2}$ of *NTL* index is increased from 0.2488 to 0.8406. *IISI* increased from 0.3015 to 0.7509; *VHNI* increased from 0.2642 to 0.8647. It can be concluded that the linear relationship between lighting index *TNL* and urban employment population is stronger than the linear relationship between regional population in southeast China. At the same time, in the economically developed southeast region, the linear regression among *VHNI* with GDP, urban employment population and power consumption at the municipal level is stronger than the other two indices.

### 3.3. Linear Regression within the Province

Southeast China, with its excellent geographical position, has taken the lead in the surging economic growth of China and become the most developed region in China’s economy. The region includes Jiangsu, Zhejiang, eight other provinces, and one city, Shanghai, whose municipality is directly under the central government. The economic development and industrial layout are different, so the contribution of different industries to GDP, population and power demand cannot be generalized. Shanghai is a municipality directly under the central government and has districts and counties under its jurisdiction, so it does not participate in the linear regression analysis. The three indicators of GDP, population and power consumption of eight provinces are analyzed one by one.

#### 3.3.1. Linear Regression between *TNL* and GDP

**Figure 10:**
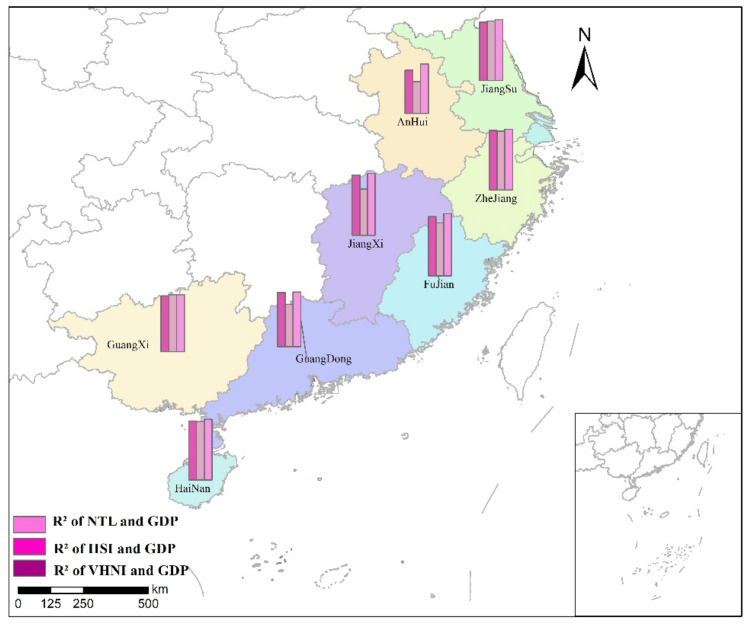
$R^{2}$ spatial distribution of *TNL* and GDP linear regression.

GDP is the GDP of each city in eight provinces, and the unit is 100 million yuan. *TNL* is the *TNL* of different indices within the administrative scope of each city. Linear regression is conducted between *TNL* of three indices in different cities and local GDP, and analysis and comparison are made. The linear regression results of GDP and *TNL* within each city in the same province are shown in Table 5. The $R^{2}$ spatial distribution of *TNL* and GDP linear regression is shown in Figure 10.

It can be seen from the regression results of the study region that the *VHNI* index has a better linear regression effect on GDP than the other two indices after regional division by province. Among many provinces, the linearity of GDP and *TNL* is the strongest in Zhejiang province, followed by Jiangsu and Hainan, and the linearity is weak in Anhui province. In *NTL* and *VHNI* indices, Jiangxi and Fujian had the best regression effect, while Anhui had the worst. The value of $R^{2}$ of *IISI* is lower than that of *NTL* and *VHNI* in most provinces, but its linear relationship is better than that of *NTL* and weaker than that of *VHNI* in Jiangsu and Guangxi. According to the $R^{2}$ value of the three indices fitting the municipal GDP divided by province, it can be seen that GDP has the best linear correlation with *VHNI* index, which is higher than other indices. The provinces with the best fitting degree of *VHNI* and GDP are Jiangxi and Fujian.

**Figure 11:**
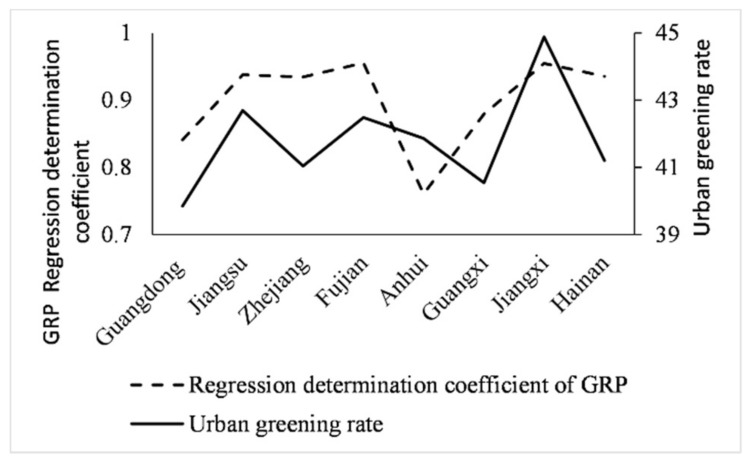
Comparison of GDP regression $R^{2}$ and urban green rate curve in southeast China.

According to the *China Urban Statistical Yearbook*, the urban greening rates of Jiangxi and Fujian are 44.88% and 42.48% respectively, ranking first among provinces in southeast China, followed by Jiangsu and Zhejiang. In order to further explore the linear regression effect of *VHNI* and GDP, the regression $R^{2}$ is compared with the urban green rate, as shown in Figure 11. From the curve change of $R^{2}$ and urban greening rate, the trend of the two is basically the same in different provinces, so there is a certain correlation between them, and the correlation coefficient is 0.4312.

#### 3.3.2. *TNL* Had a Linear Regression with Urban Employed Population

**Figure 12:**
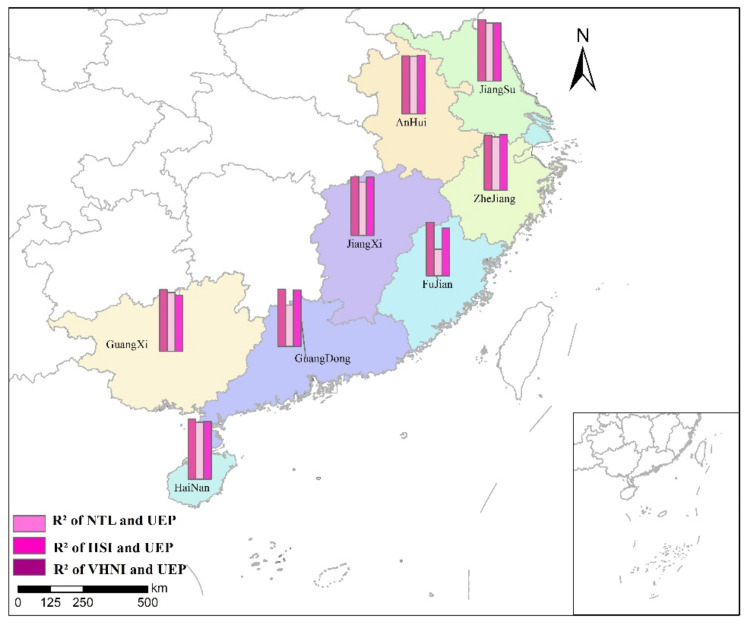
$R^{2}$ spatial distribution of *TNL* and urban employed population linear regression.

Considering the abnormal trend of the curves of the three indices and population in Figure 5 in many cities, and the good linear relationship between the urban employed population and *TNL* shown in Figure 9, the urban employed population was used to replace the number of total population for linear regression, with the unit being ten thousand. The results of linear regression between municipal *TNL* and the number of urban employed population in the same province are shown in Table 6. The $R^{2}$ spatial distribution of *TNL* and urban employed population linear regression is shown in Figure 12, where UEP is urban employed population.

Results shown in Table 6, compared with the $R^{2}$ value in Table 3, replace the index after linear fitting and significantly enhance the decision coefficient $R^{2}$ value. The overall fitting precision is greatly increased, further confirmed in the town’s southeastern employment population compared to the local population. The *TNL* have better linear relation, and the research in this area can provide additional options. For example, in the linear regression of Guangxi province, the $R^{2}$ of *NTL* is 0.8972, the $R^{2}$ of *IISI* is 0.8563 and the $R^{2}$ of *VHNI* is 0.8168. Except for Zhejiang and Anhui, the $R^{2}$ of *VHNI* is the largest in the three indices, and the $R^{2}$ of *NTL* is the largest in the other provinces. Therefore, different from GDP and power consumption as indicators of linear regression, the index with the strongest linear relationship with urban employment population is *NTL*, followed by *VHNI*, and the weakest is *IISI*.

#### 3.3.3. Linear Regression between *TNL* and Power Consumption

**Figure 13:**
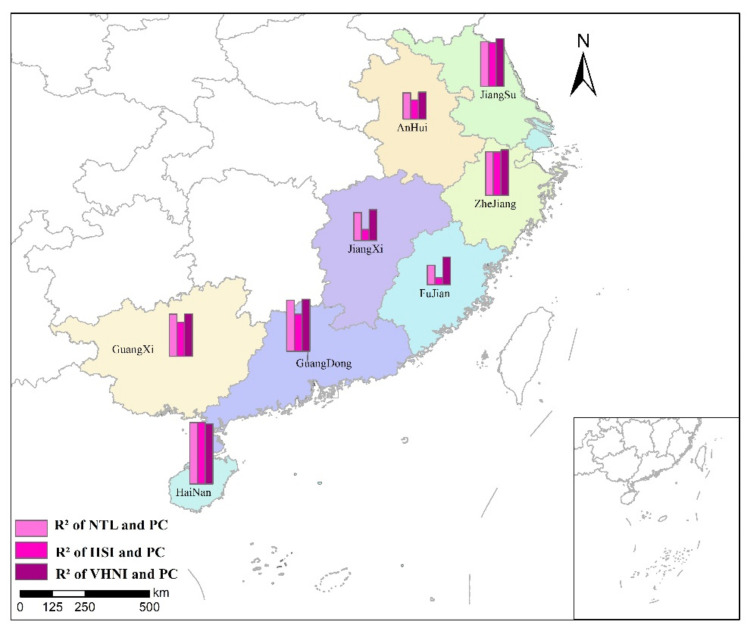
$R^{2}$ spatial distribution of *TNL* and power consumption linear regression.

The linear regression results of *TNL* of the three indices and power consumption are shown in Table 7. The $R^{2}$ spatial distribution of *TNL* and power consumption linear regression is shown in Figure 13, where PC is power consumption.

The regression effect of *VHNI* and power consumption in Hainan is the best, where $R^{2}$ is 0.9483, followed by Guangdong 0.8199, Jiangsu 0.7526 and Zhejiang 0.7196 while Anhui has the worst effect, only 0.4251. *IISI* index has the worst linear relationship with power consumption, and the overall linear relationship is weaker than *VHNI* and *NTL*, except Hainan’s $R^{2}$ is 0.9734. The correlation between *NTL* index and power consumption is between *VHNI* and *NTL*.

## 4. Discussion

In this paper, linear, logarithm and exponential models are compared to perform regression analysis on GDP, population and power consumption. The results show that, among the three models, the linear regression model has the highest correlation with the three indicators. Using the linear regression method, the nighttime light images and three kinds of social and economic statistics of 100 cities in southeast China were analyzed. The lighting data of different cities were obtained by *NTL*, *IISI* and *VHNI* indices, and the *TNL* and GDP, population and power consumption of each index were divided into overall and provincial scales for linear regression.

In the overall linear regression, *TNL* of the three indices had a strong linear relationship with GDP and power consumption, and the regression effect was good. By comparing the value of $R^{2}$ of the linear regression between the three indices and GDP, *VHNI* performed best in the regression of the three indices, $R^{2}$ was 0.8632, which was higher than *NTL* 0.8466 and *IISI* 0.7746. In the linear regression of power consumption, the $R^{2}$ of *VHNI* was 0.7824, which is higher than 0.752 of *NTL* and 0.6404 of *IISI*. However, in the linear regression between the index and population, the three indices fitted this index poorly. The $R^{2}$ of *VHNI*, the previous best performer, was only 0.2488, lower than *NTL*’s 0.2642 and *IISI*’s 0.3015. Considering the influence of many social factors in southeast China, the $R^{2}$ values of the three kinds of index regression were greatly improved after adopting urban employed population instead of regional population as the linear regression index. It shows that the linear relationship between the three indices and urban employment population is much stronger than that of regional population, which can provide an alternative index for future research. Therefore, in the population regression analysis, *VHNI* had the strongest linear relationship with the three indices.

Among the three linear regression indices using GDP as an indicator, the $R^{2}$ value of *VHNI* in each province was greater than that of *NTL* and *IISI*. The regression effect of *VHNI* in Jiangxi and Fujian was the best, reaching 0.95. The regression effect of *VHNI* in Anhui was poor, $R^{2}$ is only 0.7604. However, it was higher than *NTL* at 0.6638 and *IISI* at 0.492. At the same time, it can be seen from the $R^{2}$ of linear regression of *VHNI* and GDP and the curve change of urban green rate that the trend of *VHNI* and GDP was basically the same in different provinces, so there is a certain correlation between them. The correlation coefficient was 0.4312, which can provide reference for future research. After replacing regional population with urban employment population as regression index, the $R^{2}$ of *VHNI* in Zhejiang was 0.806, and the $R^{2}$ of Anhui was 0.852. The $R^{2}$ of *VHNI* was the largest in the three indices of the two provinces, and the $R^{2}$ of *NTL* in the other provinces was the largest. Different from the total regression analysis, *NTL* replaced *VHNI* index and became the index with the best linear relationship with urban employment population. In the linear regression experiment with power consumption as an indicator, *VHNI* was only in Hainan, $R^{2}$ was 0.9483, less than the *NTL* $R^{2}$ of 0.9694, and the other seven provinces all had indices with the best linear relationship with power consumption. Even if $R^{2}$ was less than 0.5 in Jiangxi, Fujian and Anhui provinces, it was greater than the other two indices. Therefore, in the provincial analysis, *VHNI* had the strongest linear relationship with GDP and power consumption.

In summary, the *NDVI* value in southeast China is high, and the linear relationship between the light limiting index *VHNI* and the two indicators (GDP and power consumption) under vegetation constraint is the strongest, which is stronger than the unrestricted light index *NTL* and the single light limiting index *IISI*. Therefore, in the study area with developed economy and high vegetation coverage, the existing or newly constructed light restriction index under vegetation constraint can be used as the optimal index in the regression study of light index and these two economic statistical parameters, and its effect should be better than the other two types of indices. However, in the same area, if population or population-related statistical parameters are used as indicators for linear regression research, unrestricted light index or single light restricted light index should be used for corresponding research without considering the influence of vegetation. According to the performance of the three indices, *VHNI* index can be used for regression analysis of GDP and power consumption in the future. Because of *VHNI*’s good linear relationship with economic and power consumption indicators, it can be used to predict the economy and power consumption. In terms of forecasting power consumption, we can also give relevant opinions on carbon emissions and climate issues.

## 5. Conclusions

According to the linear regression results of the three indices in the study area, compared with *IISI* and *NTL* indices, the newly constructed *VHNI* index has a better regression effect on regional economy and power consumption both in general and by province, and has broader application prospects. When population is used as an indicator and the overall linear regression is carried out with the regional population, the correlation of the three indices is weak, but when urban employment population is used as a population indicator, the linear relationship between *VHNI* index and urban employment population is the strongest. The linear relationship between *NTL* index and regional population was strongest when linear regression was performed by province.

The vegetation information data used by *VHNI* is the most widely used normalized vegetation index (*NDVI*). Other vegetation indices, such as enhanced vegetation index (EVI), can reduce the influence of soil background and atmosphere on vegetation index. The ratio vegetation index (RVI), which is sensitive to vegetation in high vegetation coverage area, and the perpendicular vegetation index (PVI), which eliminates the influence of soil background, can be effectively replaced according to the vegetation coverage of the study area. The research area of this paper is southeast China, which is the most economically developed region in China. In the future, the research can be extended to other regions of China and even across the globe for verification. In addition, *VHNI* is only compared with *NTL* and *IISI* in this paper. Other indices can be added in future studies for comparison, analysis and induction, and *VHNI* can be improved.

## Figures and Tables

**Table 1:** Specific administrative units in the study area.

Serial Number	Municipality or Province	Number of Cities
1	Shanghai	1
2	JiangSu Province	13
3	ZheJiang Province	11
4	FuJian Province	9
5	GuangDong Province	21
6	GuangXi Province	14
7	HaiNan Province	3
8	AnHui Province	16
9	JiangXi Province	11

**Table 2:** Data name and source.

Data Type	Data Name	Data Source
Light data	NPP/VIIRS	https://eogdata.mines.edu/products/vnl/
Vegetation data	MODIS *NDVI*	https://modis.gsfc.nasa.gov/
Administrative data	Vector of province and city administrative region	HTTP://WWW.NGCC.CN/NGCC/
Socioeconomic statistics	CHINA CITY STATISTICAL YEARBOOK	http://www.stats.gov.cn/tjsj/ndsj/

**Table 3:** Three indices versus three indicators in different regression models.

Index	Regression Model	GDP	Population	Power Consumption
*NTL*	Linear	0.8466	0.2488	0.752
	Logarithm	0.5791	0.2418	0.2259
	Exponential	0.5032	0.2418	0.2259
	Double logarithm	0.7994	0.2259	0.6453
*IISI*	Linear	0.7746	0.3015	0.6404
	Logarithm	0.5061	0.2985	0.2985
	Exponential	0.5417	0.2615	0.2615
	Double logarithm	0.7468	0.3059	0.5774
*VHNI*	Linear	0.8632	0.2642	0.7824
	Logarithm	0.5885	0.2463	0.4741
	Exponential	0.4592	0.2444	0.4979
	Double logarithm	0.8113	0.2196	0.6623

**Table 4:** The result of linear regression $R^{2}$ of different indices *TNL* on the number of urban employed population.

Index	Number of Employed People in Urban Areas
NTL	0.8406
IISI	0.7509
VHNI	0.8647

**Table 5:** Linear regression $R^{2}$ of *TNL* and GDP in each province.

Province	NTL	IISI	VHNI
Zhejiang Province	0.9181	0.9069	0.9343
Jiangxi Province	0.9284	0.7109	0.9546
Jiangsu Province	0.8958	0.9158	0.9375
Hainan Province	0.9066	0.8996	0.9354
Guangxi Province	0.8609	0.8792	0.8793
Guangdong Province	0.8332	0.6522	0.8411
Fujian Province	0.9138	0.816	0.9553
Anhui Province	0.6638	0.492	0.7604

**Table 6:** Linear regression $R^{2}$ between *TNL* and urban employed population in each province.

Province	NTL	IISI	VHNI
Zhejiang Province	0.7960	0.7763	0.806
Jiangxi Province	0.8527	0.7757	0.8447
Jiangsu Province	0.892	0.8439	0.8442
Hainan Province	0.8744	0.828	0.8369
Guangxi Province	0.8972	0.8563	0.8168
Guangdong Province	0.8337	0.598	0.8197
Fujian Province	0.7768	0.3868	0.693
Anhui Province	0.8426	0.8378	0.852

**Table 7:** Linear regression $R^{2}$ between *TNL* and power consumption within provinces.

Province	NTL	IISI	VHNI
Zhejiang Province	0.6895	0.6841	0.7196
Jiangxi Province	0.4408	0.174	0.4861
Jiangsu Province	0.7057	0.6896	0.7526
Hainan Province	0.9694	0.9734	0.9483
Guangxi Province	0.6664	0.5397	0.6716
Guangdong Province	0.8089	0.5874	0.8199
Fujian Province	0.3095	0.1109	0.4351
Anhui Province	0.4151	0.3032	0.4251

## Data Availability

Not applicable.
